# *Piper tectoniifolium* Kunth: A New Natural Source of the Bioactive Neolignan (−)-Grandisin

**DOI:** 10.3390/molecules27041151

**Published:** 2022-02-09

**Authors:** André M. Marques, Alexandre Siqueira da Rocha Queiroz, Elsie F. Guimarães, Ana Carolina Mafud, Paulo de Sousa Carvalho, Yvonne Primerano Mascarenhas, Thais da Silva Barenco, Pâmella Dourila N. Souza, David William Provance, José Hamilton M. do Nascimento, Cristiano G. Ponte, Maria Auxiliadora C. Kaplan, Davyson de Lima Moreira, Maria Raquel Figueiredo

**Affiliations:** 1 Departament of Natural Products, Pharmaceutical Technology Institute, Far-Manguinhos, Fiocruz, Sizenando Nabuco 100 St, Manguinhos, Rio de Janeiro 21041-250, RJ, Brazil; andrefarmaciarj@yahoo.com.br (A.M.M.); mraquelf6@yahoo.com.br (M.R.F.); 2 Health Sciences Center, Natural Produts Research Institut (IPPN), Federal University of Rio de Janeiro, Block H-1° Floor, Rio de Janeiro 21941-590, RJ, Brazil; siqueira.rj@hotmail.com (A.S.d.R.Q.); makaplan@uol.com.br (M.A.C.K.); 3 Botanical Garden Research Institute of Rio de Janeiro, Pacheco Leão 915 St, Jardim Botânico, Rio de Janeiro 22460-030, RJ, Brazil; eguimar@jbrj.gov.br; 4 Physics Institute of São Carlos, Universidade de São Paulo, Trabalhador São-Carlense, Av. n° 400, São Carlos 13566-590, SP, Brazil; carolmafud@gmail.com (A.C.M.); paulo.sousa@ursa.ifsc.usp.br (P.d.S.C.); yvonne@ifsc.usp.br (Y.P.M.); 5 Nucleus of Applied Biomedical Sciences—Federal Institute of Rio de Janeiro (IFRJ), Rio de Janeiro 20270-021, RJ, Brazil; thais.sbarenco@gmail.com (T.d.S.B.); pamdourila@gmail.com (P.D.N.S.); cristiano.ponte@ifrj.edu.br (C.G.P.); 6 Center for Technological Development in Health, Laboratory of Interdisciplinary Medical Research, Oswaldo Cruz Foundation, Rio de Janeiro 21040-361, RJ, Brazil; bill.provance@fiocruz.br; 7 Laboratory of Cardiac Electrophysiology Antonio Paes de Carvalho, Carlos Chagas Filho Institute of Biophysics—Federal University of Rio de Janeiro, Rio de Janeiro 21941-902, RJ, Brazil; jhmn@biof.ufrj.br

**Keywords:** piperaceae, lignans, medicinal plants, secondary metabolites, vascular reactivity

## Abstract

The *Piper* species are a recognized botanical source of a broad structural diversity of lignans and its derivatives. For the first time, *Piper tectoniifolium* Kunth is presented as a promising natural source of the bioactive (−)-grandisin. Phytochemical analyses of extracts from its leaves, branches and inflorescences showed the presence of the target compound in large amounts, with leaf extracts found to contain up to 52.78% in its composition. A new HPLC-DAD-UV method was developed and validated to be selective for the identification of (−)-grandisin being sensitive, linear, precise, exact, robust and with a recovery above 90%. The absolute configuration of the molecule was determined by X-ray diffraction. Despite the identification of several enantiomers in plant extracts, the major isolated substance was characterized to be the (−)-grandisin enantiomer. In vascular reactivity tests, it was shown that the grandisin purified from botanical extracts presented an endothelium-dependent vasorelaxant effect with an IC_50_ of 9.8 ± 1.22 μM and around 80% relaxation at 30 μM. These results suggest that *P. tectoniifolium* has the potential to serve as a renewable source of grandisin on a large scale and the potential to serve as template for development of new drugs for vascular diseases with emphasis on disorders related to endothelial disfunction.

## 1. Introduction

The plant kingdom is a natural complex library of bioactive molecules with a range of therapeutic potential. As such, it is in high demand in the pharmaceutical industry as a valuable source for novel drug leads and discovery [1]. Lignans represent a class of natural products derived from the oxidative coupling of two C_6_-C_3_ units, which have their origin in the shikimic acid pathway and cinnamic acid derivatives [2]. Most lignans are found in a free form in nature that can be isolated from different parts of a plant. Among these are lignans and neolignans composed of phenolic compounds that present a wide spectrum of biological activities [3,4]. Despite their wide distribution in vascular plant species, their biological functions in plants are still not fully determined [5]. A recognized natural source of many bioactive lignan and neolignan derivatives is the *Piper* species [3,6]. In Brazil numerous medicinal plants rich in lignan metabolites, including the *Piper* species, are used in alternative folk medicine for a number of ailments and physical conditions. For example, ethnopharmacological studies on the traditional use of *Piper truncatum* Vell suggested a potential use in vascular diseases that has led to the isolation and characterization of potentially active lignans with biomedical applications for hypertension [7].

A recent trend has been the investigation of plants containing the neolignan grandisin, which is a tetrahydrofuran (THF) lignan trimethoxy substituted metabolite shown to present remarkable biological activities. Phytochemical investigations described the isolation and characterization of this natural compound as one of the major secondary metabolites from the bark of *Cryptocarya crassinervia* Miq. (Lauraceaea) [8], *Piper solmsianum* [9] and *Virola surinamensis (Rol. exRottb),* Myrtaceace [10]. The most commonly isolated grandisin enantiomer is the (−)-grandisin form that is an all-*trans* THF lignan. However, many other enantiomers, such as (+)-grandisin, may also be present in lignan pools due to the presence of multiple chiral centers. An example is the isolation of (+)-grandisin from *Piper polysyphorum*, a native species from the south of China [11].

It is well known that the use of plant-derived compounds as a starting point for the development of new therapeutics is particularly challenging due to the quantities and purity necessary to permit in vitro screenings and in vivo trials. Too frequently, a target compound can only be isolated in small quantities in a laborious separation process from complex crude extracts that requires multiple steps and makes it difficult to continuously support biological assays [12]. Yet, multiple published results related to (−)-grandisin have shown a wide variety of pharmacological properties such as antitumoral [13,14,15], larvicidal [16], antimalarial [17], antinociceptive and anti-inflammatory [10], trypanocidal [9], and leishmanicidal [18] as well as being a competitive inhibitor of CYP2C9, CYP3A4/5 and the CYP450 enzyme type [19]. In view of the potential pharmacological value of this natural metabolite, it is important to identify and characterize natural sources that can meet the demands of in vitro and in vivo investigations as well as the industrial requirements for product development and fabrication. Here, we report on the potential of *P. tectoniifolium* Kunth, a native Brazilian shrub in the southeast region, to serve as a renewable botanical source of (−)-grandisin. A new HPLC-based method was developed to accompany and evaluate purified material. In addition, the absolute configuration of (−)-grandisin was determined and its pharmacological performance was confirmed.

## 2. Results and Discussion

### 2.1. Chemical Characterization of Piper Tectoniifolium as a Source of (−)-Grandisin

The present study is the first report on an HPLC validated method for the quantification of the neolignan grandisin from the species *P. tectoniifolium.* This species is an evergreen shrub of medium size that is native to Brazil, which is presented as a renewable botanical source for the preparative isolation of the tetrahydrofuran neolignan in sufficient quantities for industrial applications. Phytochemical studies led to the characterization of this metabolite as the major compound in extracts of leaves, branches, and inflorescences. From nonpolar extracts (*n*-hexane), separation procedures led to the isolation of a large amount of (−)-grandisin from leaves (968.0 mg from 8.2 g of *n*-hexane extract; 11.8%) and from branches (180.0 mg from 1.0 g of *n*-hexane extract; 18.0%). The results confirm the bioprospecting potential of the *P. tectoniifolium* species as a source for this bioactive neolignan.

**Figure 1:**
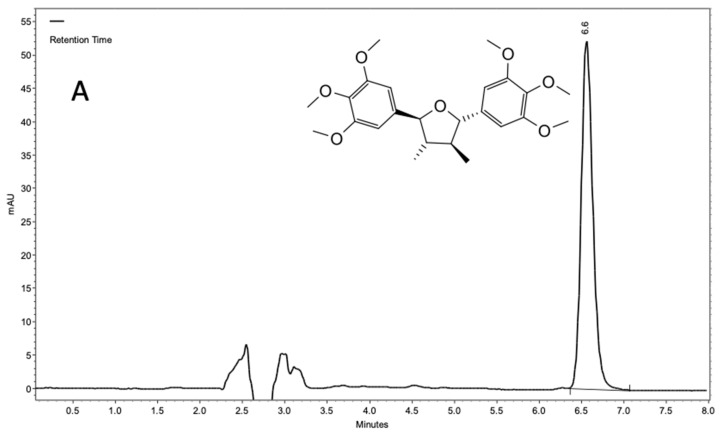
Spectra from an HPLC analysis of grandisin isolated from a methanol extract of *Piper tectoniifolium* leaves.

The HPLC-DAD-UV method developed was validated and proved to be selective for the identification of (−)-grandisin as well as sensitive, linear, precise, exact, robust and with a recovery level above 90%. The grandisin signal in the new method showed a high retention factor (α = 5.0) and signal symmetry (~1.0). No interference was observed in the chromatographic window for (−)-grandisin, even in high sample concentration (Figure 1).

The linearity of the analytical method was determined from analyses performed on three different days over the concentration range of 5, 10, 20, 30, 50, 80, 100 and 200 μg/mL, the *r* > 0.99 (*r* = 1.00000 ± 5.77 × 10^5^) without outliners and with a homoscedastic distribution. The formula to calculate (−)-grandisin in the samples was ABS (mAU) = (31,678 ± 127) concentration (μg/mL) + (11,621 ± 1344) considering the three analytical curves (Supplementary Materials, Figure S1). The variation between intraday and interday values showed that the method was precise since the RSD values were less than 15% (Supplementary Materials, Table S1). Accuracy was tested for all concentrations in the analytical curve and demonstrated a minimum value of 96.4% for 5 μg/mL and a maximum of 102.5% for 30 μg/mL. These values suggest that the method is accurate based on an allowed variance between 85 to 115% (Supplementary Materials, Table S2). For values of small parameters variation, the method proved to be robust, using up to five different changes. The parameter that demonstrated the greatest amplitude in the percentage variation coefficient was the pH (2.42% for 150 μg/mL and 1.46% for 15 μg/mL). However, the percentage variation coefficient for quantification purposes was less than 5% and therefore acceptable (Supplementary Materials, Tables S3 and S4). The analysis of standards at concentrations of 30 μg/mL and 60 μg/mL showed high recovery values of above 90% (Supplementary Materials, Table S5). The limit of detection (LOD) and quantification (LOQ), obtained by successive dilution was registered at 75 and 100 ng/mL, respectively, which are compatible with UV detection (10^−9^).

Overall, the new HPLC method can be considered validated to be sensitive for the detection and quantification of (−)-grandisin. For comparison, the published LOD and LOQ to quantify benzofuran neolignans in extracts of *Piper regnelli* were higher and in the range of 1.68 and 5.60 μg/mL, respectively [20]. Since grandisin is considered as a potential antitumoral drug candidate, previous studies have investigated the concentration of grandisin obtained from *Piper solmisianum* and from *Virola surinamensis* in biological fluids and nanoformulations, respectively [21,22]. In biological fluids, grandisin and four other metabolites were quantified with an initial retention time (Rt) of 15 min, which was optimized to 6.0 min of Rt, although the method used to determine (−)-grandisin metabolism was validated for the analysis of drugs. In the second investigation, PLGA nanocapsule formulations loaded with grandisin were evaluated with regard to the release of grandisin to improve its solubility. Despite the short Rt for grandisin, this method was not validated. The method presented here proved to be selective and displayed good resolution for grandisin characterization in complex natural matrices as crude extracts from plants. The quantification of the crude extracts of *P. tectoniifolium* with the newly developed and validated method are shown in Table 1.

The extraction of leaves with *n*-hexane transitioned a large quantity of grandisin (527.80 mg/g), which demonstrated its affinity for solvents with a low polarity. The results suggest *P. tectoniifolium* nonpolar extracts could serve as valuable sources for the isolation of (−)-grandisin on a large scale. However, the extraction from leaves should be considered since it would represent a more ecofriendly natural source to obtain the metabolite. The high quantities of (−)-grandisin in the inflorescences may be related to a chemical defense system, a hypothesis that would need to be investigated. In a comparison between methanol extracts, the amount of (−)-grandisin in leaves was 17.70 mg/g, which was less than in inflorescences (20.80 mg/g) and branches (78.40 mg/g). Again, considering an industrial procedure to obtain (−)-grandisin: while the branches showed a higher concentration with methanol, the collection of branches can lead to plant exhaustion and could make maintaining production more difficult compared to leaves. According to our results, the concentration of grandisin in the leaves and bark of *P. tectoniifolium* was higher than the published values for *Cryptocarya crassinervia* Miq. (Lauraceaea) [8], *Piper solmsianum* [9] and *Virola surinamensis (Rol. exRottb),* Myrtaceace [10] species.

### 2.2. Crystalline and Molecular Structure of (−)-Grandisin Confirmed by X-ray Diffractometry

**Figure 2:**
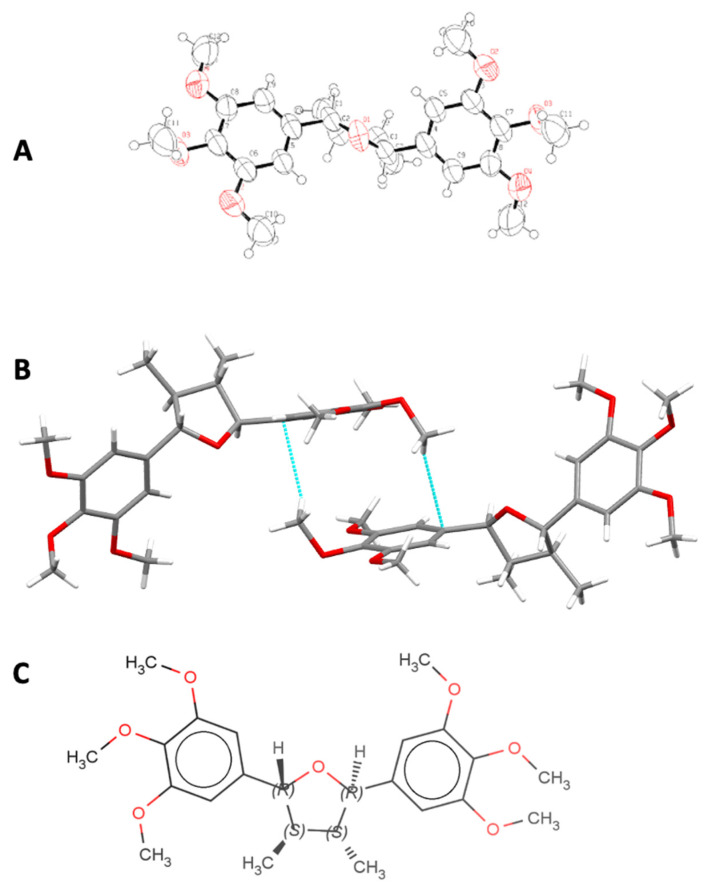
Molecular structure of (−)-grandisin. (**A**) The perspective of the asymmetric part of the molecular structure of (−)-grandisin with complete molecular structure with numbering scheme and displacement ellipsoids drawn at the 50% probability level; (**B**) perspective of the electronic interactions between (−)-grandisin molecules for the formation of the crystalline reticulum (perspective view of the C-H···π interactions (dashed cyan lines) dimer in grandisin, with graph-set R^4^_4_(12)); (**C**) absolute configuration of grandisin determined by X-ray (2R,3S,4S,5R)-3,4-dimethyl-2,5-bis(3,4,5-trimethoxycyclohexyl)oxolane.

The dihedral angle formed between the two benzene rings was verified to be 120° by X-ray diffractometry (Figure 2A). The data suggested that the crystalline structure is formed from interactions of π bonds in positions 1 and 1′ with hydrogen atoms of the methoxy groups in positions 4 and 4′ (Figure 2B). The structure of (−)-grandisin was determined as showed in Figure 2C.

However, through a critical analysis of the chromatograms and mass spectra obtained from different samples, it was possible to detect the occurrence of other substances with the same molecular weight, but in lower concentrations that are most likely grandisin isomers. This conclusion would agree with the variety of THF neolignans derived from grandisin such as all cis and all trans THF lignans found by Ramos et al. (2017), which were also found in *Piper solmsianum.* The confirmation of the corrected configuration among aryls and methyl groups presented in THF lignans such as grandisin is fundamental to expanding the understanding on stereochemical diversity and its possible effects on pharmacological assays [23].

### 2.3. Pharmacological Evaluation

**Figure 3:**
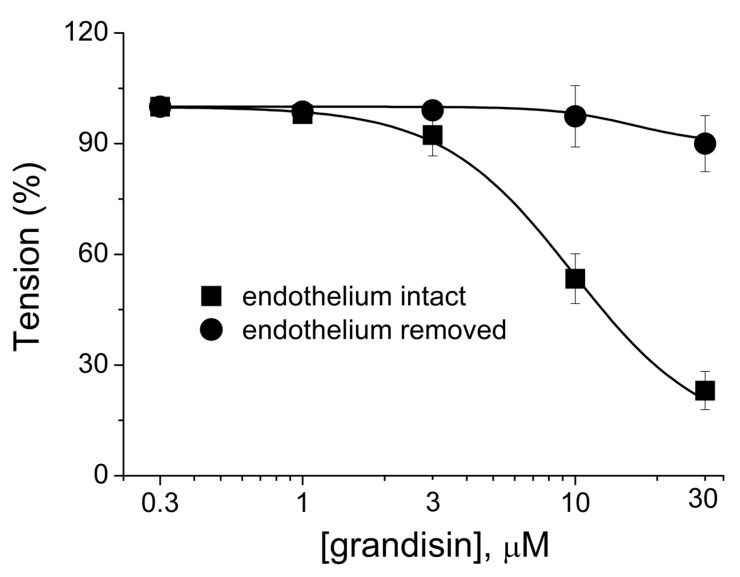
Concentration-response curves of (−)-grandisin in endothelium-intact and removed aortic rings pre-contracted with PHE 1 μM. Cumulative concentrations of (−)-grandisin (0.3 μM, 1 μM, 3 μM, 10 μM and 30 μM) were added. All data are expressed as mean ± standard error of the mean (SEM) (*n* = 5).

The previous chemical and pharmacological investigations of *P. tectoniifolium* suggested that this species was the source of an THF neolignan endowed with a vasodilating effect that still lacks studies to clarify the mechanisms of action. To examine the pharmacological activities of (−)-grandisin purified from *P. tectoniifolium*, rat aorta rings were prepared and precontracted with 1 µM phenylephrine (PHE). The concentration-dependent relaxation (IC_50_ = 9.8 ± 1.22) effect of (−)-grandisin on aorta arterial rings with their endothelium intact is shown in Figure 3 (closed boxes). It was observed that at a concentration of 30 µM, ~80% relaxation was measured. Removal of the vascular endothelium considerably abolished the relaxation caused by (−)-grandisin (Figure 3, closed circles), which suggests that the vascular effect of (−)-grandisin acts on endothelium cells.

Studies with THF lignans from other *Piper* species such as (−)-cubebin isolated from *P. cubeba* L. have also demonstrated a vasorelaxant effect. However, the effect appeared to be irrespective of the presence of endothelium. Carvalho et al. (2013) showed that (−)-cubebin was able to reduce PHE contraction in intact rings, suggesting that the THF lignan promotes vasorelaxation via the NO/cGMP pathway in rat aorta without prostacyclin involvement [24]. In another study, Raimundo et al. (2009) demonstrated the vasodilatory activity of crude extracts of leaves of *P. truncatum* Vell. as well as the pure isolated lignan eudesmin, suggesting the possible therapeutical properties of these natural products for the treatment of hypertension according to traditional medicine. In this case, nitric oxide release and the involvement of the histamine receptor in the endothelial cells were related to the observed biological effects [7].

Endothelial dysfunction is one of the most relevant features in the pathophysiology of hypertensive disorders such as hypertension. Substances that directly affect endothelial functional regulation such as (−)-grandisin studied in the present paper may have significant potential in the treatment of circulatory diseases [25]. Plant-derived natural products can be useful for drug discovery development, especially when they are based on previous traditional knowledge. However, bioprospection from botanicals can be very challenging, depending on plant species and the concentration of biological markers. Since plant sources are often slow growing and tend to accumulate target compounds at very low quantities over long growth periods or in specific climate conditions, it is of great importance to identify natural sources that can supply the scale needed to meet market demands [12]. Our results from extracts of different parts of *P. tectoniifolium* Kunth strongly suggest that this plant could be a renewable source to produce (−)-grandisin on a large scale that could allow the development of new treatments for hypertension.

## 3. Materials and Methods

### 3.1. Chemicals

All solvents, i.e., technical grade n-hexane, chloroform, methanol, acetonitrile and HPLC grade acetonitrile and methanol were provided by TEDIA, Brazil. MilliQ deionized water was obtained at the laboratory and acidified with glacial acetic acid (Merck, Brazil).

### 3.2. Botanical Material

The species *Piper tectoniifolium* Kunth was collected by Dr. Maria Raquel Figueiredo in Matipó City (20°17′44.6″ S 42°21′28.5″ W), Minas Gerais State, Brazil, in March 2014. The botanical material was identified by the botanist Dr. Elsie Franklin Guimarães and deposited at the Herbarium RB of the Botanical Garden Research Institute of Rio de Janeiro, under number RB606977. This work was carried out with the CGEN license number AB5D82.

### 3.3. Extract Preparation and Separation Procedures

Dried plant material consisting of branches (260 g), leaves (232 g) and inflorescences (85 g) were maintained separate and reduced to small fragments. The plant materials were separately extracted by static maceration with *n*-hexane, methanol over three days with daily solvent exchange.

#### 3.3.1. Separation Procedures

##### (−)-Grandisin Isolation by Precipitation

**Figure 4:**
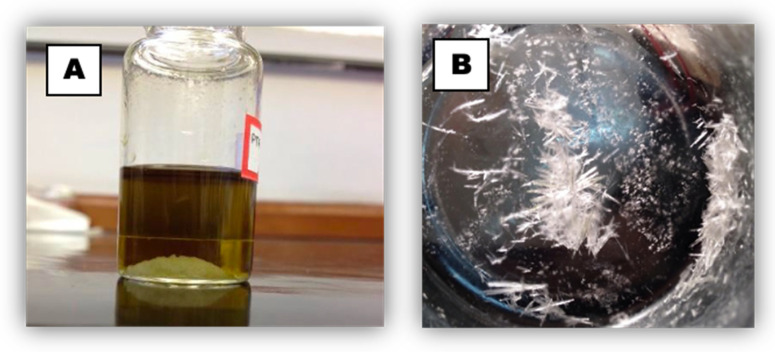
Precipitation from an *n*-hexane extract of branch material: (**A**) precipitated material after transfer of the primary extract into the solvent chosen for the countercurrent chromatography (CCC) system; (**B**) recrystallized material after 24 h in contact with the CCC solvent system.

A portion of an *n*-hexane extract obtained from branches was prepared to be submitted to countercurrent chromatography separation (CCC). The chromatographic profile chosen was composed of a mixture of *n*-hexane (Hex)/ethyl acetate (EtOAc)/MeOH/H_2_O at a 4:2:2:0.5 ratio. When the sample (1.0 g) was introduced into the chosen solvent system, a precipitate of white crystals in the bottom of the flask was observed (Figure 4A). To favor further precipitation in a slow and uniform manner, the flask was covered with a watch glass. After 24 h, the precipitate was observed to form needle-like crystals (Figure 4B). The remaining solvent was removed leaving only semipurified crystals yielding 180 mg.

##### (−)-Grandisin Isolation by Silica Gel Chromatographic Column

Adsorption chromatography on a silica gel column was utilized to process an *n*-hexane extract from leaves of *P. tectoniifolium* (8.2 g). The components of the extract were eluted from the column by solvent systems prepared with binary mixtures of Hex, EtOAc and MeOH in a gradient of increasing polarities that resulted in 63 fractions. Based on similar TLC profiles, fractions 39–50 that eluted from the column in concentrations ranging from 20–50% (*n*-Hex/EtOAc) were pooled (2.0 g). The pooled fractions were subjected to a second fractionation process also using a silica gel column that yielded 120 fractions. Through the comparison with the chromatographic standard previously obtained, it was possible to confirm by TLC presence of grandisin in fractions 41–63. The fractions enriched with grandisin were next subjected to exclusion chromatography on a Sephadex LH-20 column using a 3:1 mixture of methanol/chloroform. This procedure led to a yield of ~968.0 mg of grandisin with relative purity above 94.0% (analyzed by GC-FID and GC-MS).

### 3.4. GC-FID Analysis

Quantitative and qualitative analyses were performed on a GC 2010 Shimadzu equipped with a ZB-1MS fused silica capillary column (30 m × 0.25 mm i.d. × 0.25 μm film thickness). The operating temperatures were 260 °C at the injector, 290 °C at the detector and from 60 °C to 290 °C at 10 °C/min in a column oven. Hydrogen was used as the carrier gas at a constant flow of 1.0 mL/min. The percent purity of the compound was obtained from the peak areas of the GC-FID analysis.

### 3.5. GC-MS Analysis

Qualitative analyses were performed on a GC-QP2010 PLUS Shimadzu equipped with a ZB-5MS fused silica capillary column (30 m × 0.25 mm i.d. × 0.25 μm film thickness). The operating temperatures were injector, 270 °C; detector, 290 °C; and from 60 °C up to 290 °C at 3 °C/min in the column oven. Helium (99.99%) was used as the carrier gas at a constant flow at 1.0 mL/min for GC coupled to mass spectrometer (MS) analysis. The target compound was identified by comparison of its mass spectra (see Supplementary Materials) with published data [9].

### 3.6. NMR Analysis

Nuclear magnetic resonance spectra of hydrogen and carbon (^13^C and ^1^H NMR) were obtained on Varian device VNMRS-Gemini 500 spectrometer operating at a frequency of 400 MHz (^1^H)/100 MHz (^13^C) using CD_3_OD as the solvent. Special techniques and bidimensional measurements such as COSY, HMBC, HSQC were also performed. The chemical shift values (δ) in dimensionless units were referred to an internal standard (TMS), being represented in parts per million (ppm) of the applied frequency for each experiment and coupling constants (*J*) were measured in Hz. The obtained data (see Supplementary Materials) were compared to literature data [9].

### 3.7. Quantification of the Major Isolated Lignan (−)-Grandisin from Extracts of P. tectoniifolium

The quantification of (−)-grandisin was performed at the Laboratory of Environmental Toxicology of the Oswaldo Cruz Foundation. For the quantification of (−)-grandisin, a Shimadzu^®^ CLASS-VP High-Performance Liquid Chromatograph coupled to a Diode Array Ultraviolet detector (HPLC-DAD-UV) was used. Additional equipment included a controller (SCL 10A VP), degasser (DGU-14A), binary pump (LC-10AD VP), oven (CTO-10AS VP) and detection system (DAD SPD M10A VP). Chromatograms were analyzed using the Schimadzu Class VP^®^ program version 6.1. All runs were performed with a Thermo ODS Hypersil column (250 mm × 4.6 mm i.d. × 5 µm, particle size) using a mobile phase in the isocratic mode with a 65/35 mixture of acetonitrile/MilliQ deionized water acidified with glacial acetic acid (pH 3.0) at a flow rate of 1.0 mL/min and an oven temperature at 50 °C for detection at 234 nm. The injector washing solvent was methanol. The total analysis time was 8 min with an average pressure of 76 bar. Under these conditions, the retention time (rt) of (−)-grandisin was registered to be 6.6–7.0 min. Standard and sample injection volume was 20 mL.

This original method was developed and validated for the analysis of grandisin in extracts of *P. tectoniifolium* in relation to selectivity, linearity, precision, accuracy, limit of detection (LOD) and quantification (LOQ), robustness and recovery in accordance with DOQ-CGCRE-008—V2 from the National Standards for Analytical Validation of The National Institute of Metrology, Standardization and Industrial Quality/Brazil [26]. A stock solution of grandisin in methanol at 200 mg/mL was prepared on the day of the analysis to generate solutions of 5, 10, 20, 30, 50, 80 and 100 μg/mL. *Linearity* was determined from these concentrations over three different days. *Accuracy* was determined to represent the degree of agreement between the determinations obtained by the method under study in relation to a value accepted as a reference and which was also tested for those concentrations. *Precision* was expressed as the standard deviation (SD) or relative standard deviation (RSD) from measurements of 15 and 150 μg/mL obtained on three different days (inter-day) as well as morning and afternoon runs (intraday). The *robustness* of an analytical method is a measure of its ability to resist small and deliberate variations in the analytical parameters, being indicative of reliability during normal use of the instrument. This parameter was tested in relation to acetonitrile in the mobile phase, flow rate, pH, temperature and acid type. *Recovery* measures the efficiency of the extraction procedure for an analytical method. Recovery tests were performed by spiking 30 or 60 μg/mL of grandisin standard solution into the samples. LOD and LOQ were obtained by serial dilution and analysis of the relation of signal to noise that is 1/3 and 1/10, respectively. All analysis was performed in triplicate from solution of the extracts and fractions from *P. tectoniifolium.*

### 3.8. X-ray Diffractometry

A single crystal grandisin was analyzed by X-ray diffraction to determine the absolute configuration of the molecule, considering that it has four stereogenic centers. Data collections was in a KappaCCD Nonius diffractometer, Mo radiation, crystal system hexagonal, space group P6522. The structure was solved by direct methods and revealed that in the grandisin, [C_24_H_32_O_7_], the asymmetric unit is composed by half molecule with the oxygen O1 in a special position, on a rotation axis, *n* = 2, and the symmetry operation {x, −y, −z} gives rise to the complete molecule. After refinement the absolute configuration of the molecule was confirmed by the low value of the Flack parameter.

### 3.9. Pharmacological Evaluation

Animals were anesthetized by isoflurane (1% mg/kg) inhalation, and after the loss of motor control, rats were euthanized by cervical dislocation. Subsequently, the thoracic aorta was dissected and cleansed of connective tissue to obtain rings of 2 mm in length that were suspended in a 5 mL organ bath (myograph model 620M, DMT, Denmark). Tissues were kept at 37 °C in a Krebs Henseleit buffer (120 mM NaCl, 5 mM KCl, 1.1 mM MgCl_2_, 2.5 mM CaCl_2_, 1.2 mM NaH_2_PO_4_, 10 mM *N*-[2-hydroxyethyl] piperazine-*N*’-[2-ethane-sulfonic acid] (HEPES), 15 mM NaHCO_3_ and 11 mM glucose; pH 7.4) that was constantly bubbled with a gas mixture of 95% O_2_ and 5% CO_2_. Each arterial segment was suspended between two steel hooks connected to an isometric transducer to measure tension through a data acquisition system (PowerLab 8 and LabChart Pro, AD-Instruments, Australia). After a stabilization period of 60 min at a rest tension of 1.0 g with periodic changes of solution (every 15 min), a stable contraction was achieved with 1 μM phenylephrine (PHE). Functional endothelial integrity was assessed by the ability of 10 μM acetylcholine to induce ≥70% relaxation in vessels precontracted with 1 μM PHE [27]. For vascular reactivity tests, the rings were preconstricted with 1 µM of PHE and after a stable response, the concentrations (µM) of 0.3, 1.0, 3.0, 10.0 and 30.0 of (−)-grandisin were subsequently added to the chamber and the level of relaxation measured. Values were expressed as mean ± standard error of mean (SEM). The IC_50_ values (defined as the concentration of the test compound that reduced 50% of the maximal contraction) were obtained by an actual concentration-response curve fitting using GraphPad Prism 6.0 software (GraphPad Software, San Diego, CA, USA).

#### Ethics

All animal studies (male Wistar rats (200–300 g) were carried out in strict accordance with the guidelines approved by the Animal Use Ethics Committee from the Federal University of Rio de Janeiro (CEUA/CCS/UFRJ 087/15).

## 4. Conclusions

For the first time, the evergreen shrub species *P. tectoniifolium* has been presented as a natural alternative source for grandisin, a bioactive metabolite with several pharmacological activities with an emphasis toward disorders related to endothelial disfunction. The medicinal interest for this neloginan in drug discovery strategies led to our development of a new HPLC method that was validated for its analysis in plant extracts. The method was also useful for quality control and to obtain fingerprints of various botanical medicines. The highest concentration of the target compound was identified in nonpolar extracts of leaves (≈53%), which is a suitable amount for mass production. Considering that it is a renewable source, it would be of interest to conduct further research on phytochemical bioprospection strategies to enhance the yields of secondary metabolites such as (−)-grandisin from nonpolar leaf extracts of *P. tectoniifolium*. Together, the results suggest that the botanical source, methods and yields have the potential to provide the metabolite for development of new drugs for vascular diseases.

## Figures and Tables

**Table 1:** Quantification of (−)-grandisin in different vegetative parts and extracts of *Piper tectoniifolium*.

Sample *	(−)-Grandisin Average %	SD	RSD%	mg/g	SD
*n*-Hexane leaf extract	52.78	0.52	0.98	527.80	5.20
Methanol leaf extract	1.77	0.02	1.35	17.70	0.20
Methanol branch extract	7.84	0.03	0.45	78.40	0.30
Methanol inflorescence extract	2.08	0.00	0.20	20.80	0.04

* For extract preparation, see material and methods.

## Data Availability

All data can be found in supplementary materials as supporting data.

## Supplementary Materials

The following are available online, Figure S1: analytical curves obtained in three different days in the range of 5 to 200 μg/mL, Figure S2: HPLC chromatogram for the sample grandisin obtained from nonpolar extract of *P. tectoniifolium* leaves, Figure S3: GC-MS chromatogram for the sample grandisin, obtained from *P. tectoniifolium* leaves extract, Figure S4: mass profile of the grandisin, Figure S5: ^1^H NMR spectrum of grandisin neolignan, Figure S6: ^13^C NMR spectrum of grandisin neolignan, Figure S7: data referring to ^1^H (400 MHz) and ^13^C (100 MHz) NMR spectra in CDCl3 of grandisin neolignan isolated from *Piper tectoniifolium*, Figure S7: COSY ^1^H × ^1^H Homonuclear Correlation Spectrum for grandisin neolignan, Figure S8: HMBC ^1^H × ^13^C Heteronuclear Multiple Bond Correlation Spectrum for the grandisin neolignan, Figure S9: HSQC ^1^H × ^13^C Heteronuclear Correlation Spectrum for the grandisin neolignane, Table S1: intraday and interday precision, values of absorbance in mAU, Table S2: accuracy test in the range 5 to 200 μg/mL of standard solution of (−)-grandisin, Table S3: robustness parameters, Table S4: robustness experimental tests for 15 and 150 μg/mL. Table S5: Robustness experimental tests for 15 and 150 μg/mL. Table S6: Data referring to 1H (400 MHz) and 13C (100 MHz) NMR spectra in CDCl3 of grandisin neolignan isolated from Piper tectoniifolium.
