# Supplementary material for: Piper tectoniifolium Kunth: A New Natural Source of the Bioactive Neolignan (−)-Grandisin
Source: Molecules. 2022 Feb 9;27(4):1151. doi: 10.3390/molecules27041151 (PMC8876808; doi:10.3390/molecules27041151)
Supplement: Supplementary file 1 [file molecules-27-01151-s001.zip › molecules-1553480-supplementary.pdf]

Supplementary material for:

Piper tectoniifolium **Kunth: a new natural source of the neolignan (-)-grandisin with potential vascular effects**

André M. Marques[1], Alexandre Siqueira da Rocha Queiroz[2], Elsie F. Guimarães[3], Ana Carolina Mafud[4], Paulo de Sousa Carvalho Jr[4], Yvonne Primerano Mascarenhas[4], Thais da S. Barenco[5], Pâmella Dourila N. Souza[5], D. William Provance Jr.[6], José Hamilton M. do Nascimento[7], Cristiano G. Ponte[5], Maria Auxiliadora C. Kaplan[2], Davyson de Lima Moreira[3*], Maria Raquel Figueiredo[1]

Supplementary material:

Quantification of (-)-grandisin in different vegetative parts and extracts of *Piper tectoniifolium*.

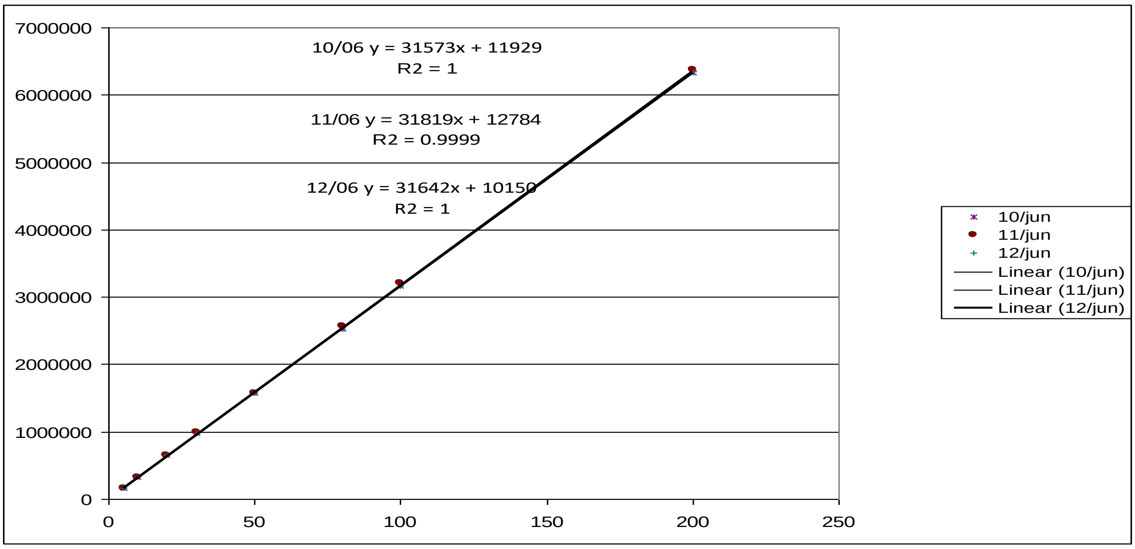

Figure S1 - Analytical curves obtained in three different days in the range of 5 to 200 µg/ mL.

Table S1 - Intra-day and inter-day precision. Values of absorbance in mAU.

**Intra-day* 15 µg/mL**	**AVR**	**Intra-day* 150 µg/mL**	**AVR**	**Inter-day* 15 µg/mL**	**AVR**	**Inter-day* 150 µg/mL**	**AVR**
	490913		4943103		492539		4943103
**SD**	2020	**SD**	20698	**SD**	2808	**SD**	20698
**RSD %**	0.41	**RSD %**	0.42	**RSD %**	0.57	**RSD%**	0.42

* six repetition of the same concentration in the same day (intra-day) or in different days (inter-days); AVR = average; SD = standard deviation; RSD % = relative standard deviation.

Table S2 - Accuracy test in the range 5 to 200 μg/mL of standard solution of (-)-grandisin.

Nominal [ ]μg/mL	Abs 1	Abs 2	Abs 3	Abs Average	[ ] calc	Δμg	Δ% μg	Δ%
200	6331978	6371797	6347893	6350556	200.11	0.11	0.05	100.05
100	3167927	3204472	3166183	3179527	100.00	0.00	0.00	100.00
80	2528831	2570371	2524594	2541266	79.85	-0.15	-0.18	99.82
50	1568422	1569367	1580622	1572804	49.28	-0.72	-1.43	98.57
30	983332	990435	984319	986028.9	30.76	0.76	2.53	102.53
20	652740	653226	649271	651745.4	20.21	0.21	1.04	101.04
10	327377	327542	326729	327216	9.96	-0.04	-0.37	99.63
5	163593.3	165258	164138	164329.7	4.82	-0.18	-3.59	96.41

[ ] = concentration; [ ] calc = concentration of (-)-grandisin calculated from de analytical curve; Abs = absorbance in mAU; D = difference.

Table S3 - Robustness parameters.

	Nominal		Variation		number
**ACN in Mobile Phase %**	65	A	66	a	**1**
**Flow Rate**	1	B	0.9	b	**2**
**pH**	3.0	C	3.5	c	**3**
**Temperature Oven**	50	D	45	d	**4**
**Acid Type**	acetic	E	phosphoric	e	**5**

ACN = acetonitrile (HPLC grade, Tedia, Brazil); acetic and phosphoric acids from Sigma-Aldrich (Brazil).

Table S4 – Robustness experimental tests for 15 and 150 μg/mL.

Parameters	0	1	2	3	4	5
**ACN in Mobile Phase %**	A	**a**	A	A	A	A
**Flow Rate**	B	B	**b**	B	B	B
**pH**	C	C	C	**c**	C	C
**Temperatura oven**	D	D	D	D	**d**	D
**Acid type**	E	E	E	E	E	**e**
**150 μg/mL**	4958537	4968870	5151483	5234026	5063866	5208143
	4977901	4990776	5137569	5283034	5058509	5203008
	4977078	4983942	5149535	5044924	5016598	5168147
**Average**	**4971172**	**4981196**	**5146196**	**5187328**	**5046324**	**5193099**
**SD**	**10950**	**11208**	**7534**	**125736**	**25883**	**21761**
**RSD%**	**0.22**	**0.23**	**0.15**	**2.42**	**0.51**	**0.42**
**15 μg/mL**	501268	499852	502577	502890	496497	509927

	501032	492796	504001	516983	491126	500105
	493051	493813	504557	505974	493126	500957
**Average**	498450	495487	503712	508616	493583	503663
**SD**	4677	3814	1021	7409	2715	5441
**RSD%**	0.94	0.77	0.20	1.46	0.55	1.08

ACN = acetonitrile (HPLC grade, Tedia, Brazil); acetic and phosphoric acids from Sigma-Aldrich (Brasil). 0 = nominal condition (acetonitrile 65%; flow rate 1 mL.$^{-1}$; pH = 3.0; temperature oven 50 ^{o}C; acid type = acetic); 1 = (**acetonitrile 66%**; flow rate 1 mL.$^{-1}$; pH = 3.0; temperature oven 50 ^{o}C; acid type = acetic); 2 = (acetonitrile 65%; **flow rate 0.9 mL.$^{-1}$**; pH = 3.0; temperature oven 50 ^{o}C; acid type = acetic); 3 = (acetonitrile 65%; flow rate 1 mL.$^{-1}$; **pH = 3.5**; temperature oven 45 ^{o}C; acid type = acetic); 4 = (acetonitrile 65%; flow rate 1 mL.$^{-1}$; pH = 3.0; **temperature oven 45 ^{o}C**; acid type = acetic); 5 = (acetonitrile 65%; flow rate 1 mL.$^{-1}$; pH = 3.0; temperature oven 50 ^{o}C; **acid type = phosphoric**).

Table S5 – Robustness experimental tests for 15 and 150 µg/mL.

Sample	Average	SD	100%	SD	Recovery %
Methanol leaf extract (10 mg/mL) + grandisin (60 µg/mL)	4209300	3788	4210260	2105	99.98
Methanol leaf extract (10 mg/mL) + grandisin (30 µg/mL)	3239765	5831	3241688	3890	99.94

Supplementary material:

Chemical characterization of (-)-grandisin isolated from *Piper tectoniifolium*.

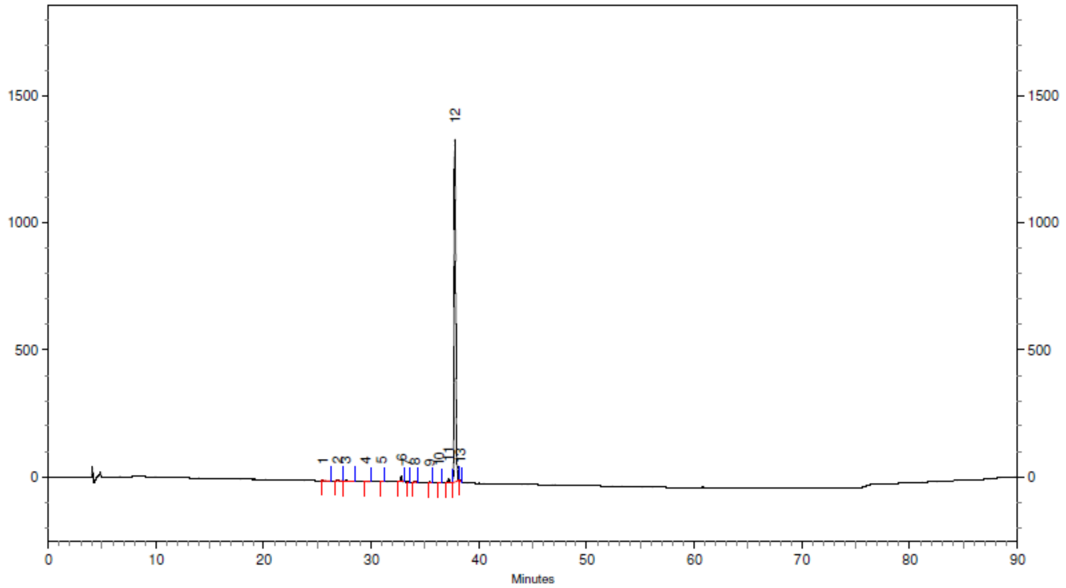

Figure S2 - HPLC chromatogram for the sample grandisin obtained from the dichloromethane extract of *P. tectoniifolium* leaves.

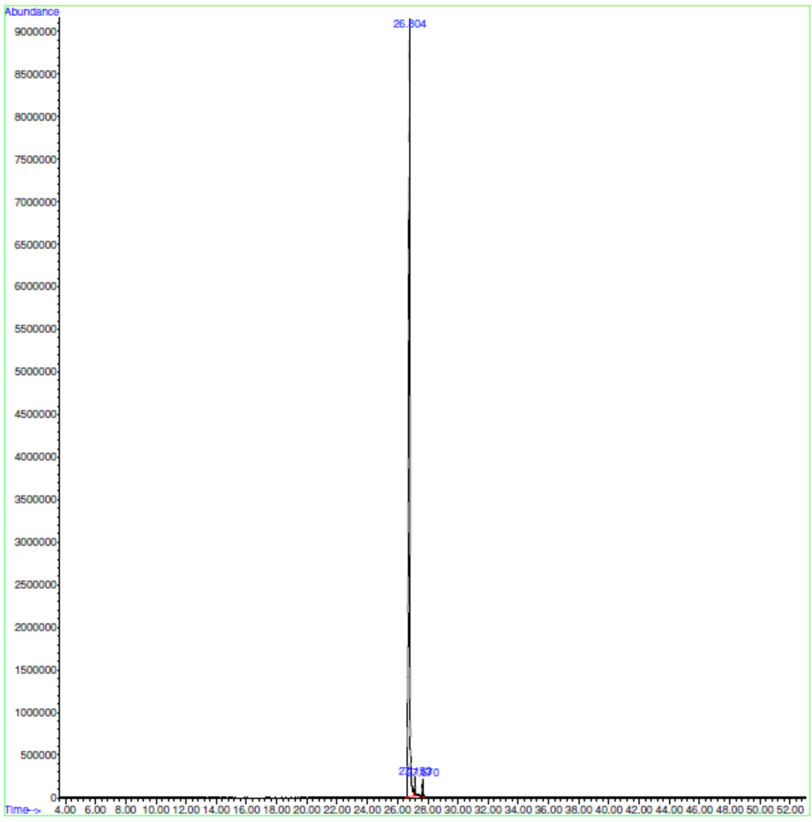

Figure S3 - GC-MS chromatogram for the sample grandisin, obtained from *P. tectoniifolium* leaves extract.

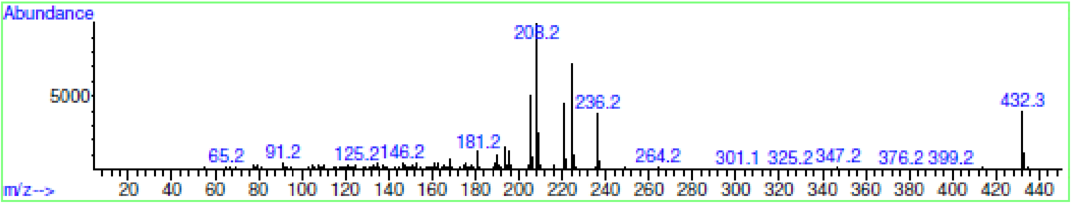

Figure S4 - Mass profile of the grandisin.

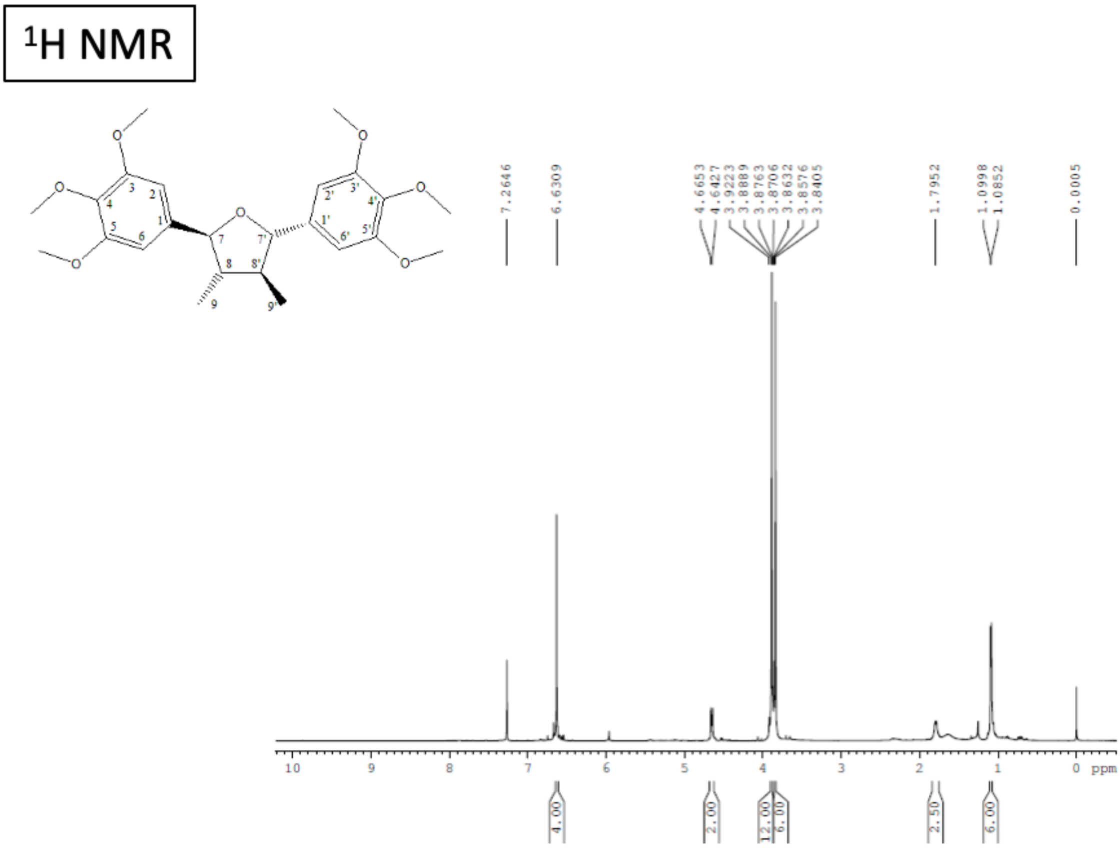

Figure S5 - ^{1}H NMR spectrum of grandisin neolignan.

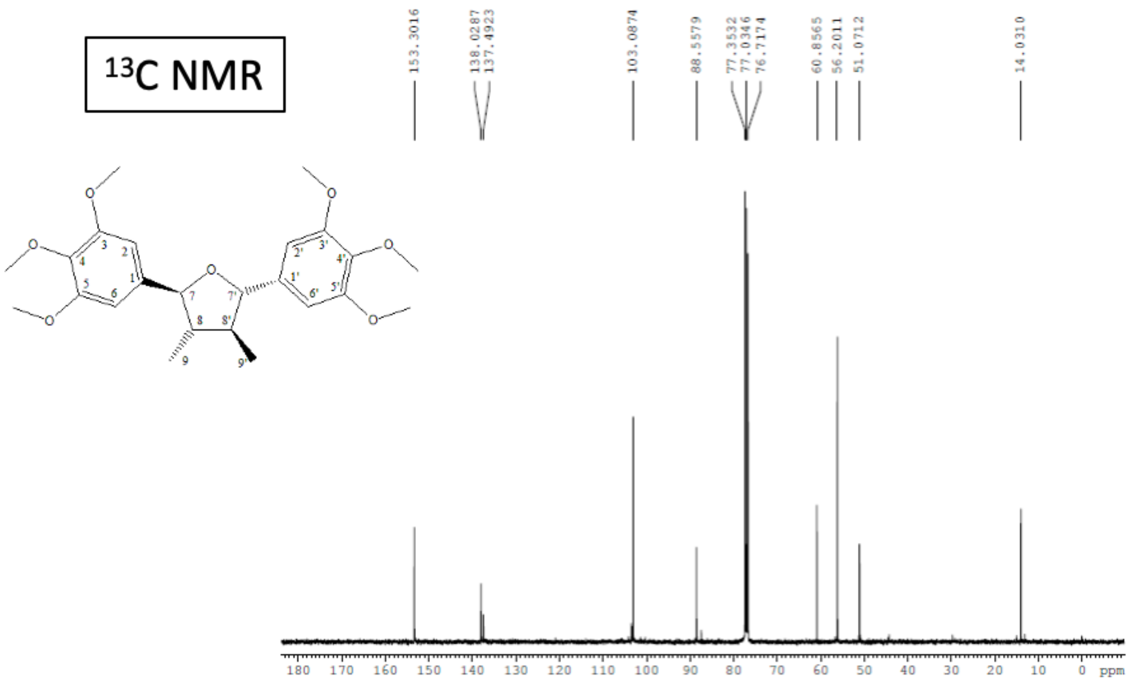

Figure S6 - ^{13}C NMR spectrum of grandisin neolignan.

Table S6- Data referring to ^{1}H (400 MHz) and ^{13}C (100 MHz) NMR spectra in $CDCl_3$ of grandisin neolignan isolated from *Piper tectoniifolium*.

**Position**	**^{1}H, δ Grandisin**	**^{13}C, δ Grandisin**
1 e 1′	-	137.5
2 e 2′	6.63, *s*	103.1
3 e 3′	-	153.3
4 e 4′	-	138.0
5 e 5′	-	153.3
6 e 6′	6.63, *s*	103.1
7 e 7′	4.67, *d*	88.6
8 e 8′	1.80, *m*	51.1
9 e 9′	1.10, *d*	14.0
MeO-3 e MeO-3′	3.89, *s*	56.2
MeO-4 e MeO-4′	3.84, *s*	60.9
MeO-5 e MeO-5′	3.89, *s*	56.2

*δH multiplicity (J in Hz); *s* = singlet, *d* = doublet, *m* = multiplet.

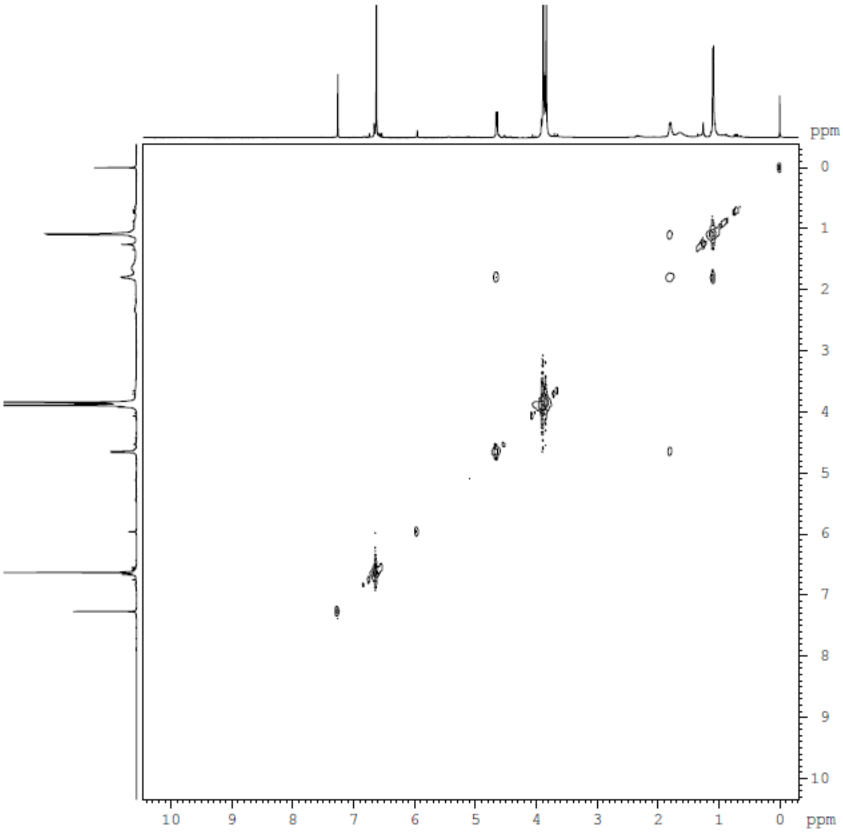

Figure S7 - COSY ^{1}H x ^{1}H Homonuclear Correlation Spectrum for grandisin neolignan.

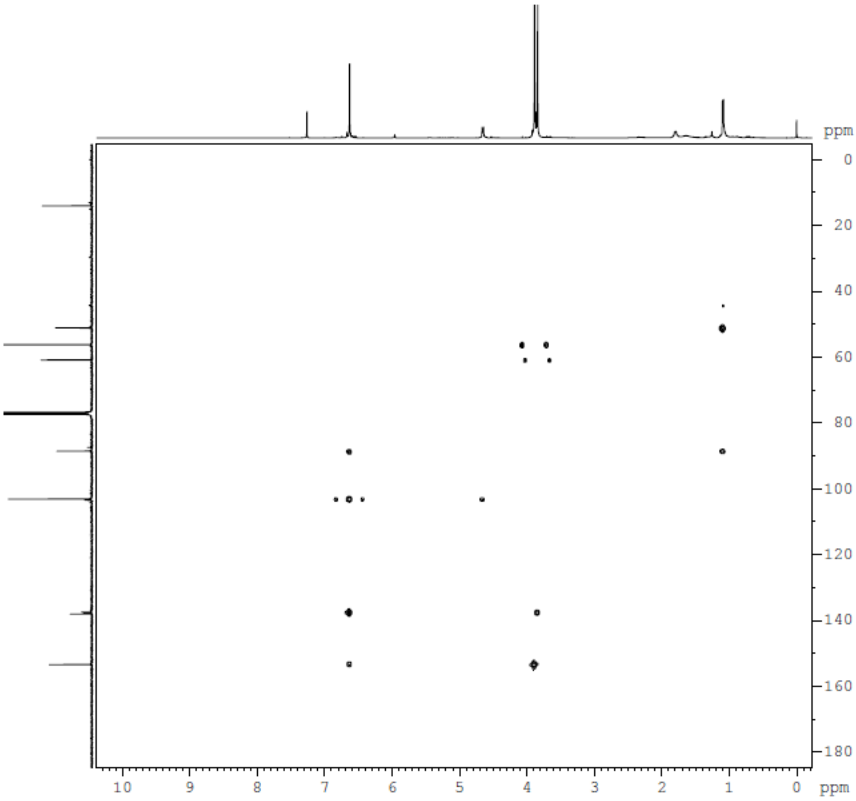

Figure S8 - HMBC ^{1}H x ^{13}C Heteronuclear Multiple Bond Correlation Spectrum for the grandisin neolignan.

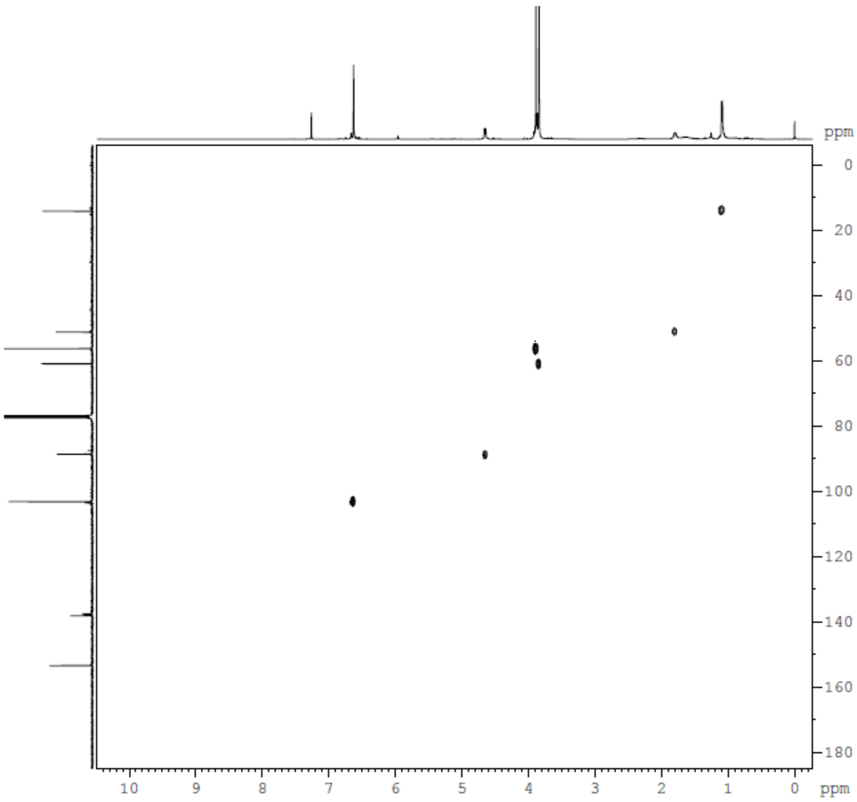

Figure S9 - HSQC ^{1}H x [13]C Heteronuclear Correlation Spectrum for the grandisin neolignan.
